# GPR40/GPR120 Agonist GW9508 Improves Metabolic Syndrome-Exacerbated Periodontitis in Mice

**DOI:** 10.3390/ijms25179622

**Published:** 2024-09-05

**Authors:** Yanchun Li, Hong Yu, Maria F. Lopes-Virella, Yan Huang

**Affiliations:** 1 Division of Endocrinology, Diabetes and Metabolic Diseases, Department of Medicine, College of Medicine, Medical University of South Carolina, Charleston, SC 29425, USA; liyya@musc.edu (Y.L.); virellam@musc.edu (M.F.L.-V.); 2 Department of Biomedical & Community Health Sciences, The James B. Edwards College of Dental Medicine, Medical University of South Carolina, Charleston, SC 29425, USA; yuho@musc.edu

**Keywords:** fatty acid receptors, periodontitis, metabolic syndrome, inflammation

## Abstract

G protein-coupled receptor (GPR)40 and GPR120 are receptors for medium- and long-chain free fatty acids. It has been well documented that GPR40 and GPR120 activation improves metabolic syndrome (MetS) and exerts anti-inflammatory effects. Since chronic periodontitis is a common oral inflammatory disease initiated by periodontal pathogens and exacerbated by MetS, we determined if GPR40 and GPR120 activation with agonists improves MetS-associated periodontitis in animal models in this study. We induced MetS and periodontitis by high-fat diet feeding and periodontal injection of lipopolysaccharide, respectively, and treated mice with GW9508, a synthetic GPR40 and GPR120 dual agonist. We determined alveolar bone loss, osteoclast formation, and periodontal inflammation using micro-computed tomography, osteoclast staining, and histology. To understand the underlying mechanisms, we further performed studies to determine the effects of GW9508 on osteoclastogenesis and proinflammatory gene expression in vitro. Results showed that GW9508 improved metabolic parameters, including glucose, lipids, and insulin resistance. Results also showed that GW9508 improves periodontitis by reducing alveolar bone loss, osteoclastogenesis, and periodontal inflammation. Finally, in vitro studies showed that GW9508 inhibited osteoclast formation and proinflammatory gene secretion from macrophages. In conclusion, this study demonstrated for the first time that GPR40/GPR120 agonist GW9508 reduced alveolar bone loss and alleviated periodontal inflammation in mice with MetS-exacerbated periodontitis, suggesting that activating GPR40/GPR120 with agonist GW9508 is a potential anti-inflammatory approach for the treatment of MetS-associated periodontitis.

## 1. Introduction

Periodontitis, a bacteria-induced chronic inflammatory disease, is characterized by progressive destruction of tooth-supporting connective tissue and periodontal alveolar bone, leading to tooth loss [1,2]. It has been well-established that diabetes exacerbates periodontitis [3]. Interestingly, clinical studies have further demonstrated that metabolic syndrome (MetS), which is commonly associated with prediabetes, also increases the prevalence and severity of periodontitis [4,5,6,7,8,9]. MetS is defined as a cluster of cardiovascular risk factors, including obesity, high blood pressure, lipid abnormalities such as high triglycerides and low HDL, insulin resistance, and increased plasma glucose [10,11]. A recent systematic review and meta-analyses showed a dose-response relationship between the number of cardiovascular risk factors in MetS and the occurrence of periodontitis [9]. Another meta-analysis also showed that MetS is positively associated with the severity of periodontitis [12].

The findings of the relationship between MetS and periodontitis are important since 34% of the US population have MetS as contrasted to 9.3% of the US population having diabetes [13]. Given that MetS worsens periodontitis, it is important to carefully evaluate and effectively treat periodontitis in patients with MetS as patients with diabetes. Indeed, early treatment of periodontitis in MetS patients who also have prediabetes is likely to reduce the severity of periodontitis when prediabetes progresses to diabetes [14]. More importantly, patients with MetS may also have life-threatening complications such as cardiovascular disease [15], nonalcoholic fatty liver disease [16], and Alzheimer’s disease [17], and increasing studies have shown that periodontitis worsens these MetS complications via periodontitis-promoted systemic inflammation [18,19,20]. Therefore, the development of novel and effective therapeutics for MetS-exacerbated periodontitis is essential to reduce not only tooth loss but also the severity of MetS and MetS complications.

To study the pathogenesis of MetS-associated periodontitis, our lab has developed several mouse models with both MetS and periodontitis [21,22,23]. In these animal models, MetS was induced by high-fat diet (HFD) feeding for 20 weeks, and periodontitis was induced by periodontal LPS injection, ligature placement around the tooth, or oral inoculation of *Porphyromonas gingivalis* [21,22,23]. Using these animal models, we demonstrated that MetS worsened periodontitis by increasing macrophage infiltration and the production of inflammatory cytokines in periodontal tissue and promoting osteoclastogenesis, which led to alveolar bone loss [21,22]. Obviously, MetS, as a systemic disorder, worsens periodontitis by augmenting periodontal inflammation. Hence, we hypothesized that compounds that are capable of not only improving MetS but also inhibiting tissue inflammation may have the potential to treat periodontitis in patients with MetS as an adjunctive therapy to scaling and root planning (SRP), the gold standard treatment of chronic periodontitis [24,25,26]. By combining anti-inflammatory therapy with SRP, the treatment of periodontitis in patients with MetS can be more effective.

In recent years, it has been well established that G protein-coupled receptor (GPR)40 and GPR120 as fatty acid (FA) receptors play important roles in not only uptaking FAs but also mediating anti-inflammatory signaling [27,28,29]. It has been shown that GRP40 or GPR120 activation in animal models improved inflammation-associated metabolic disorders such as diabetes and MetS [28,30]. It has also been shown that omega-3 (ω-3) FAs such as docosahexaenoic acid (DHA) and eicosapentaenoic acid (EPA) as natural agonists for both GPR40 and GPR120 [31,32] have beneficial effects on periodontitis [33,34]. To increase potency and specificity, several synthetic agonists for GPR40 or GPR120, as well as dual agonists for both GPR40 and GPR120, have been developed [35,36,37,38]. A large number of studies have shown that these agonists have therapeutic potential for many chronic diseases, including MetS and atherosclerosis, through anti-inflammatory mechanisms [32,39,40]. Since GPR40/GPR120 agonists are capable of not only improving MetS but also acting as potent anti-inflammatory agents, they may have beneficial effects on MetS-exacerbated periodontitis. However, whether activation of GPR40/GPR120 by agonists improves MetS-associated periodontitis remains unknown. In this study, we hypothesized that GPR40/GPR120 dual agonist GW9508 is capable of inhibiting the progression of periodontitis exacerbated by MetS. Our specific objectives include the following: 1. To determine the effect of GW9508 on alveolar bone loss and periodontal inflammation in a mouse model for MetS-associated periodontitis; 2. To explore the potential molecular mechanisms involved in GW9508-attenuated alveolar bone loss and periodontal inflammation.

## 2. Results

### 2.1. GW9508 Treatment Improves MetS

**Figure 1:**
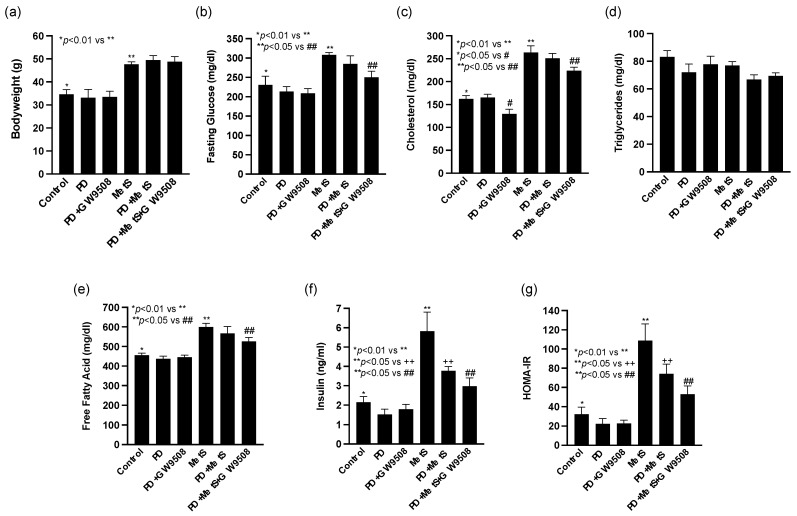
The effects of GW9508 on the metabolic parameters for control (LFD-fed) or MetS (HFD-fed) mice with or without LPS-induced periodontitis (PD). Eight-week-old male C57BL/6 mice were fed LFD or HFD for 20 weeks and periodontally injected with PBS or LPS in the last 4 weeks. Some of the mice were treated with GW9508 by intraperitoneal injection during the last 4 weeks. At the end of the experiment, the metabolic parameters, including bodyweight (**a**), fasting glucose (**b**), cholesterol (**c**), triglycerides (**d**), free fatty acids (**e**), insulin (**f**), and HOMA-IR (**g**) were determined. The data are presented as mean ± SD (*n* = 6). Group symbols: * Control, ** MetS, ++ PD+MetS, # PD+GW9508, ## PD+MetS+GW9508.

We first determined the effects of GW9508 on HFD-induced MetS. As shown in Figure 1, compared with control mice that were fed a low-fat diet (LFD), MetS mice that were fed HFD had increased bodyweight, fasting glucose, cholesterol, free fatty acid, insulin, and homeostatic model assessment for insulin resistance (HOMA-IR). Periodontitis (PD) induced by periodontal LPS injection had no significant effect on these metabolic parameters in LFD-fed control mice but reduced insulin and insulin resistance in HFD-fed mice. Interestingly, GW9508 treatment significantly reduced MetS-increased fasting glucose, cholesterol, and free fatty acids (FFA) in mice, suggesting that GW9508 as a GPR40/GPR120 agonist improves MetS.

### 2.2. GW9508 Reduces Alveolar Bone Loss in Mice with MetS-Associated Periodontitis

**Figure 2:**
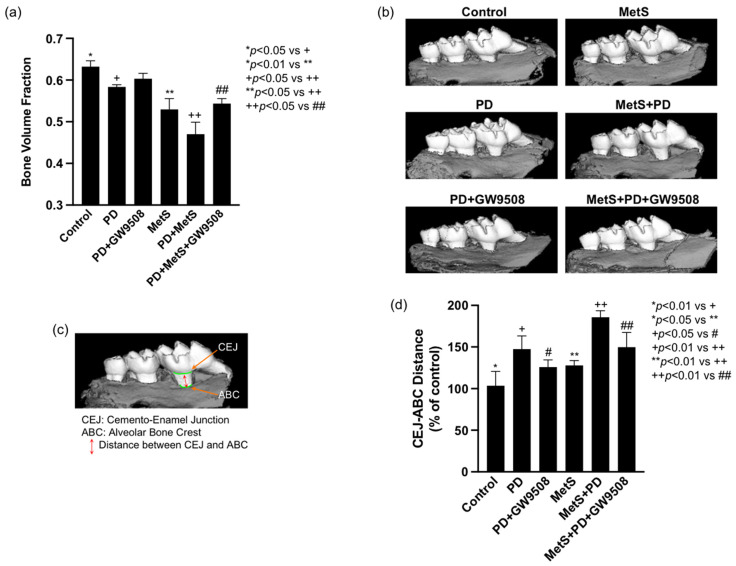
The effect of GW9508 on alveolar bone loss in mice with periodontitis (PD) alone or with MetS and PD. Male C57BL/6 mice were fed LFD or HFD and periodontally injected with LPS. After the treatment, the maxillae were scanned by mCT, and bone volume fraction was quantified (**a**,**b**). Additionally, the distance from CEJ to ABC was measured for the first and second molars (**b**–**d**). The data are presented as means ± SD (*n* = 6). Group symbols: * Control, ** MetS, + PD, ++ PD+MetS, # PD+GW9508, ## PD+MetS+GW9508.

At the end of the animal experiment, the mouse maxillae were dissected and scanned by micro-computed tomography (μCT) for bone volume fraction (BVF) quantification. As shown in Figure 2a and Table 1, periodontitis or MetS alone reduced BVF significantly, and the combination of periodontitis and MetS further reduced BVF. In contrast, GW9508 significantly reversed the loss of BVF in mice with both periodontitis and MetS. We also measured the distance between the cemento–enamel junction (CEJ) and alveolar bone crest (ABC) since CEJ–ABC distance is positively associated with alveolar bone loss [22]. Results showed that periodontitis or MetS significantly increased CEJ–ABC distance, and the combination of periodontitis and MetS further increased CEJ–ABC distance. Consistent with the findings from the mCT study, GW9508 significantly reduced CEJ–ABC distance in mice with both periodontitis and MetS (Figure 2b–d).

### 2.3. GW9508 Inhibits Osteoclastogenesis in Mice with MetS-Associated Periodontitis

**Figure 3:**
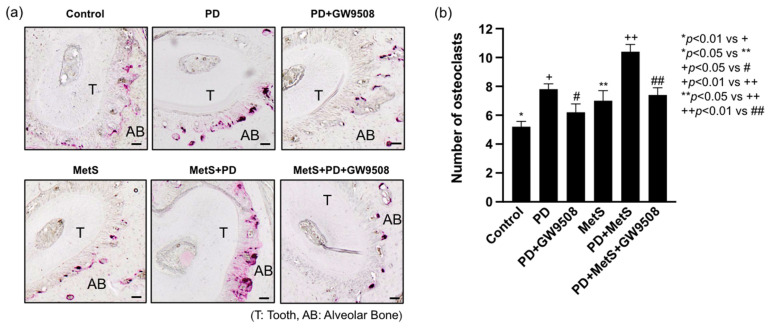
The effect of GW9508 on osteoclastogenesis in mice with periodontitis (PD) alone or with MetS and PD. After the maxillae were examined by mCT, as described in Figure 2, they were decalcified and sectioned. Tartrate-resistant acid phosphatase (TRAP) staining to detect osteoclasts was performed on the tissue sections. Representative tissue images from 6 groups of mice were shown (scale bar = 100 μm) (**a**), and multinucleated TRAP-positive osteoclasts per field of view were quantified and compared among the groups (**b**). The data are presented as mean ± SD (*n* = 6). Group symbols: * Control, + PD, # PD+GW9508, ** MetS, ++ PD+MetS, ## PD+MetS+GW9508.

To understand how GW9508 reduced alveolar bone loss in mice with MetS-associated periodontitis, we performed Tartrate-resistant acid phosphatase (TRAP) staining on tissue sections of the maxillae to detect osteoclasts. We focused on the area of periodontal ligaments which connects the tooth root and alveolar bone. Results showed that periodontitis or MetS increased osteoclast formation on the surface of alveolar bones, and the combination of periodontitis and MetS further increased osteoclast formation (Figure 3a,b). However, GW9508 treatment significantly reduced the number of osteoclasts in mice with periodontitis and MetS (Figure 3a,b).

### 2.4. GW9508 Inhibits Osteoclastogenesis Enhanced by LPS Plus Palmitic Acid (PA) In Vitro

**Figure 4:**
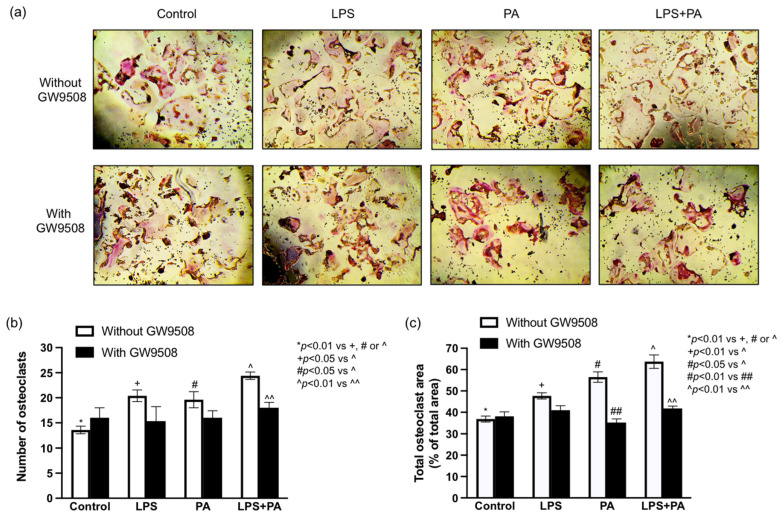
The effect of GW9508 on osteoclast formation stimulated by LPS, PA, or both LPS and PA. RAW264.7 macrophages were treated with 100 ng/mL of RANKL alone (control) or with RANKL plus 1 ng/mL of LPS, 100 mM of PA, or both LPS and PA for 3 days. After changing the medium, the macrophages were treated with RANKL alone or RANKL plus LPS, PA, or LPS and PA in the absence or presence of 1 mM of GW9508 for 2 days, and cells were then fixed. TRAP staining was carried out, and the osteoclasts, which were positively stained and contained more than three nuclei, were identified (**a**), counted, and compared among different groups (**b**). The total osteoclast area was also quantified as % of the total area and compared among different groups (**c**). The data are presented as mean ± SD (*n* = 3). Group symbols: * Control cells without GW9508 treatment, + LPS treatment without GW9508, # PA treatment without GW9508, ## PA treatment with GW9508, ^ LPS+PA without GW9508, ^^ LPS+PA with GW9508.

In this study, we used a receptor activator of nuclear factor kappa-B ligand (RANKL) to induce osteoclast formation from RAW264.7 macrophages and determined the effect of GW9508 on osteoclast formation. Since we have found previously that LPS and palmitic acid (PA), a saturated fatty acid, cooperatively enhanced RANKL-induced osteoclast formation in vitro [22], we also determine the effect of GW9508 on osteoclast formation enhanced by LPS, PA, or LPS plus PA. Consistent with our previous observations [22], results showed that while either LPS or PA increased osteoclast number and area, the combination of LPS and PA further augmented osteoclast number and area (Figure 4a–c). Results also showed that GW9508 treatment significantly inhibited the osteoclast number and area enhanced by LPS plus PA in vitro (Figure 4a–c), which is consistent with the findings from our animal study shown in Figure 3.

### 2.5. GW9508 Reduces Periodontal Inflammation in Mice with MetS-Associated Periodontitis

**Figure 5:**
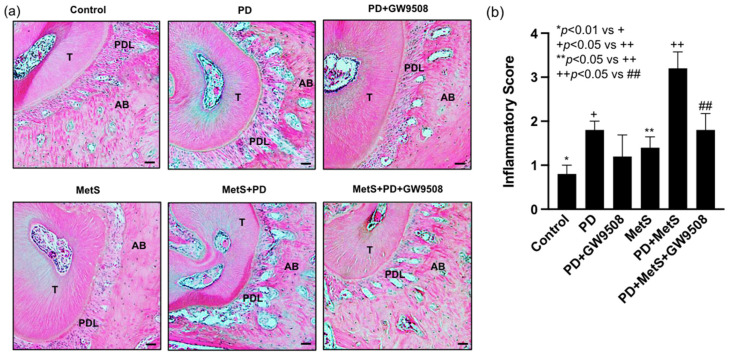
The effect of GW9508 on periodontal inflammation and bone resorption in mice with periodontitis (PD) or with MetS and PD. The periodontal tissue sections were stained with hematoxylin and eosin, and histologic analysis was performed. Representative photomicrographs focused on the area of periodontal ligament (PDL) along with tooth root (T) and alveolar bone (AB) were shown (scale bar = 100 μm) (**a**). The inflammatory scoring for leukocyte infiltration and bone resorption in all groups were made (**b**) according to the criteria as described in Methods. The data are presented as means ± SD (*n* = 6). Group symbols: * Control, + PD, ** MetS, ++ PD+MetS, ## PD+MetS+GW9508.

Histological analysis of maxillae with a focus on the area of periodontal ligaments between the tooth root and alveolar bone was performed, and inflammatory scoring was made according to the criteria described in Materials and Methods to assess the effect of GW9508 on periodontal inflammation and bone resorption in mice. Results showed that while periodontitis increased the inflammatory scores as evidenced by increased leukocyte infiltration and bone resorption, the combination of MetS and periodontitis further increased the inflammatory score in mice (Figure 5a,b). In contrast, GW9508 treatment reduced the inflammatory scores in mice with MetS-associated periodontitis as determined by decreased leukocyte infiltration and bone resorption (Figure 5a,b).

### 2.6. GW9508 Inhibits Proinflammatory Cytokine Secretion from Macrophages In Vitro

**Figure 6:**
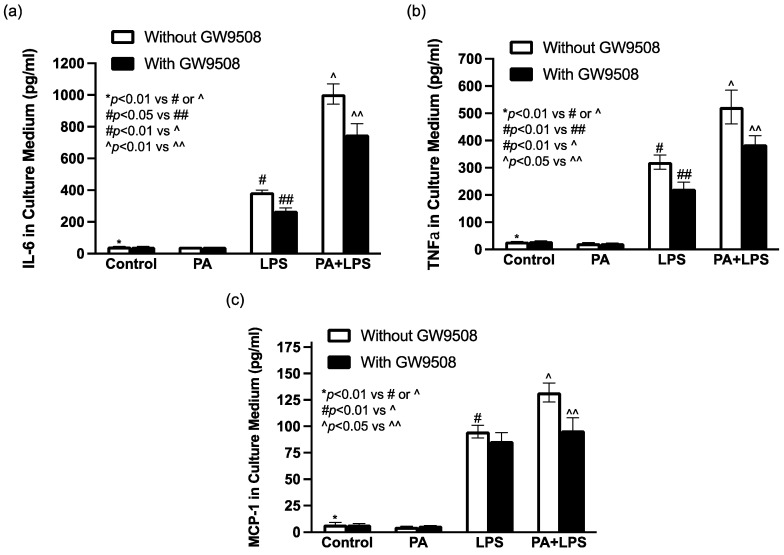
The effect of GW9508 on proinflammatory cytokine secretion from macrophages. RAW264.7 macrophages were treated with 1 ng/mL of LPS, 100 μM of palmitic acid (PA), or both LPS and PA in the absence or presence of 1 μM of GW9508 for 24 h. After the treatment, IL-6 (**a**), TNFa (**b**), and monocyte chemoattractant protein (MCP)-1 (**c**) in a culture medium were quantified with ELISA. The data presented as means ± SD are from one of three experiments with similar results. Group symbols: * Control cells without GW9508, # PA treatment without GW9508, ## PA treatment with GW9508, ^ LPS+PA without GW9508, ^^ LPS+PA with GW9508.

To explore the mechanisms by which GW9508 reduced periodontal inflammation in mice, we tested our hypothesis that GW9508 inhibits the secretion of proinflammatory cytokines from macrophages, which are immune cells known to play a pivotal role in periodontal inflammation [41]. Since LPS is released by Gram-negative bacteria that play a critical role in periodontitis, while PA is the most common saturated fatty acid in humans [42] and associated with MetS [43], we determined the effect of GW9508 on inflammatory cytokine secretion from macrophages stimulated by LPS and PA. We have shown previously that LPS and PA have a synergy on proinflammatory cytokine upregulation in macrophages [44]. Results showed that while LPS potently induced secretion of IL-6, tumor necrosis factor (TNF)a, and monocyte chemoattractant protein (MCP)-1, PA augmented the LPS-induced secretions (Figure 6). However, GW9508 significantly inhibited the cytokine secretion stimulated by LPS and PA (Figure 6).

## 3. Discussion

GPR40 and GPR120 are G protein-coupled receptors for medium- and long-chain FAs [45,46]. It has been well documented that GPR40 is highly expressed in pancreatic β cells, and its activation enhances glucose-induced insulin secretion [47]. It has been further shown that GPR40 is involved in metabolic homeostasis [48]. In addition to pancreatic β cells, GPR40 is also expressed by many other types of cells, such as macrophages [49], osteoclasts [50], vascular endothelial cells [51,52], and hepatocytes [53], and has anti-inflammatory properties [54,55,56,57,58]. Park et al. showed that treatment of bone marrow-derived macrophages with GPR40 agonist TAK875 or AMG1638 suppressed the activation of the NLRP3 inflammasome by IL-1b and IL-18 via NFkB and sarco/endoplasmic reticulum Ca^2+^-ATPase [59]. Our lab also showed recently that loss of GPR40 in mice worsened MetS-associated periodontal inflammation and that GPR40 agonists downregulated proinflammatory molecules in macrophages [23].

Similar to GPR40, GPR120 is also capable of promoting glucose homeostasis through incretin-dependent insulin secretion [60]. Furthermore, studies have shown that GPR120 is expressed by several types of immune cells, such as macrophages and CD4^+^ T cells [61,62]. GPR120 activation suppresses proinflammatory cytokine expression and enhances anti-inflammatory cytokine expression [63]. All the above findings suggest that GPR40 or GPR120 agonists may have favorable effects on diabetes or MetS and its inflammation-related complications.

The findings that DHA and EPA, as natural dual agonists of GPR40 and GPR120, exert anti-inflammatory effects are consistent with the beneficial role of GPR40 and GPR120 in inflammatory diseases [31,64,65,66]. DHA and EPA have similar potencies (pEC_50_) to GPR40 and GPR120 [32]. In addition to anti-inflammatory properties, DHA and EPA also have favorable effects on lipid metabolism and blood pressure [39]. To increase the specificity and potency, synthetic agonists for GPR40 or GPR120 and dual agonists for both GPR40 and GPR120 have been developed [37,67]. Interestingly, studies have suggested that the simultaneous activation of both GPR40 and GPR120 with a dual agonist may be more therapeutically useful than the activation of only one of them with a specific agonist. Satapati et al. reported that a dual agonist of GPR40 and GPR120 achieved superior glycemic control compared with a GPR40 selective agonist by improving insulin resistance in ob/ob mice [38], and it has been well documented that periodontitis and insulin resistance have a bidirectional relationship [68]. Furthermore, Huang et al. demonstrated that GW9508, a dual agonist of GPR40 and GPR120, has a dual effect of inhibiting osteoclastogenesis and promoting osteogenesis [69], which is closely related to periodontal health.

GW9508 is a well-characterized dual agonist with pEC_50_ of 7.32 and 5.46 for GPR40 and GPR120, respectively [70]. Studies have well documented that GW9508 is anti-inflammatory [55] and thereby exerts beneficial effects on many inflammation-related diseases, including nonalcoholic steatohepatitis [71], Alzheimer’s disease [72], bacterial infection [73], and atherosclerosis [32,39,40]. In the current study, we reported for the first time that GW9508 also improved periodontitis in animal models with MetS by alleviating alveolar bone loss and periodontal inflammation.

Given that MetS exacerbates periodontitis, we first investigated the effect of GW9508 on HFD-induced MetS in this study. Results showed that GW9508 reduced metabolic parameters, including fasting glucose, cholesterol, and FFAs, clearly indicating that GW9508 improves MetS. These findings are consistent with the previous reports that GW9508 alleviates hyperglycemia [74], hyperlipidemia [71], and insulin resistance [75] in various animal models. Since it is known that MetS-related cardiovascular risk factors, including hyperglycemia, hyperlipidemia, and insulin resistance, are associated with periodontitis [21], the above results have revealed an important mechanism by which GW9508 mitigates MetS-associated periodontitis by modulating metabolic parameters.

In addition to MetS, we also investigated the effect of GW9508 on osteoclastogenesis. Results showed that GW9508 treatment strongly inhibited osteoclast formation in mice with periodontitis and MetS, indicating that GW9508 reduces alveolar bone loss by inhibiting osteoclastogenesis. To further elucidate the molecular mechanism whereby GW9508 reduced alveolar bone loss in mice with both periodontitis and MetS, we used an in vitro cell model to demonstrate that GW9508 inhibited osteoclastogenesis induced by RANKL and enhanced by LPS and PA. It is well known that LPS is increased by periodontitis [76], and PA is associated with MetS [77]. Furthermore, we demonstrated previously that LPS and PA had a cooperative effect on osteoclastogenesis [22]. Therefore, the findings from the above in vitro study indicate that the inhibition of the LPS-PA cooperation on osteoclastogenesis by GW9508 is likely a molecular mechanism involved in GW9508-reduced alveolar bone loss in mice with MetS-associated periodontitis.

In this study, we further assessed the effect of GW9508 on periodontal inflammation since it is well-known that periodontal inflammation is critically involved in osteoclastogenesis [76,78]. To test our hypothesis that GW9508 attenuated the periodontal inflammation in mice with both periodontitis and MetS, we performed histological analysis on the area of the periodontal ligament and ligament-connected tooth root and alveolar bone. Results showed that GW9508 treatment reduced leukocyte infiltration in the area in mice with both periodontitis and MetS. We also performed an in vitro study to demonstrate that GW9508 inhibited the secretion of proinflammatory cytokines such as IL-6, TNFa, and MCP-1 from macrophages stimulated by LPS and PA. Since it is well-known that proinflammatory cytokines are potent stimulators of osteoclast precursor proliferation, osteoclast formation, and bone resorption in vivo [79,80], these findings suggest that the inhibition of proinflammatory cytokine secretion by GW9508 plays a pivotal role in GW9508-reduced osteoclastogenesis and subsequent alveolar bone loss.

In the animal models for human periodontitis, the oral bacterial gavage model, periodontal bacteria or LPS inoculation model, and ligature model are commonly used mouse models [81]. All these mouse models are good models for human periodontitis [81] and were used in our research [23,82]. In the current study, we utilized the periodontal LPS inoculation model for the following reasons. We have reported previously that MetS exacerbates periodontal inflammation and bone loss in a mouse model with periodontitis induced by periodontal LPS inoculation [21]. We have also reported that LPS and PA have a synergistic stimulation on the expression of proinflammatory cytokines in macrophages [44]. In the current study, based on the above findings, we hypothesized that GW9508 attenuates the progression of periodontitis in animal models and the inhibition of the synergistic effect of LPS and PA on proinflammatory cytokine expression by GW9508 is a mechanism involved in the attenuation of periodontitis progression by GW9508. To be consistent with our hypothesis, we used the periodontal LPS inoculation model in the current study.

A limitation of this study is that only male mice were used since it is known that female C57BL/6 mice are protected against HFD-induced MetS [83]. However, clinical studies have shown that the incidence of MetS in women is similar to that in men [84]. Although it is known that periodontitis has a documented higher prevalence in men (56.4%) compared to women (38.4%) [85], the incidence of 38.4% in women is still very high. Therefore, it is important to determine the effect of GW9508 on MetS-related periodontitis in both male and female mice to understand how targeting GPR40/GPR120 affects MetS-associated periodontitis in animal models with different genders. The findings will help the development of relevant gender-based treatment options for patients with MetS-associated periodontitis. To address the effect of GW9508 on MetS-associated periodontitis in female animals, LDL receptor-deficient (LDLR −/−) mice may be used as an animal model with MetS [86,87]. Wall et al. reported that female LDLR −/− mice fed an HFD developed a MetS phenotype, which is similar to that in male mice [86]. These female mice had increased bodyweight, blood glucose, glucose intolerance, and lipids, including triglycerides and cholesterol.

Similar to GPR40 and GPR120, CD36, also called fatty acid translocase (FAT), is also a receptor for long-chain free fatty acids [88]. However, CD36 is different from GPR40 and GPR120 as it is a multifunctional membrane receptor involved in macrophage foam cell formation, oxidative stress, tissue degradation, and apoptosis [88]. We have shown previously that CD36 expression in periodontal tissue is upregulated by MetS in mouse models of both periodontitis and MetS and upregulated in vitro by LPS and saturated fatty acids such as PA in macrophages [89]. Since CD36 is markedly upregulated by PA, PA is likely to bind to CD36 to stimulate the expression of proinflammatory molecules such as IL-6, MCP-1, and TNFa in macrophages [90,91]. We have also shown that PA and LPS have a synergy in activating pro-inflammatory signaling and upregulating pro-inflammatory gene expression [44].

**Figure 7:**
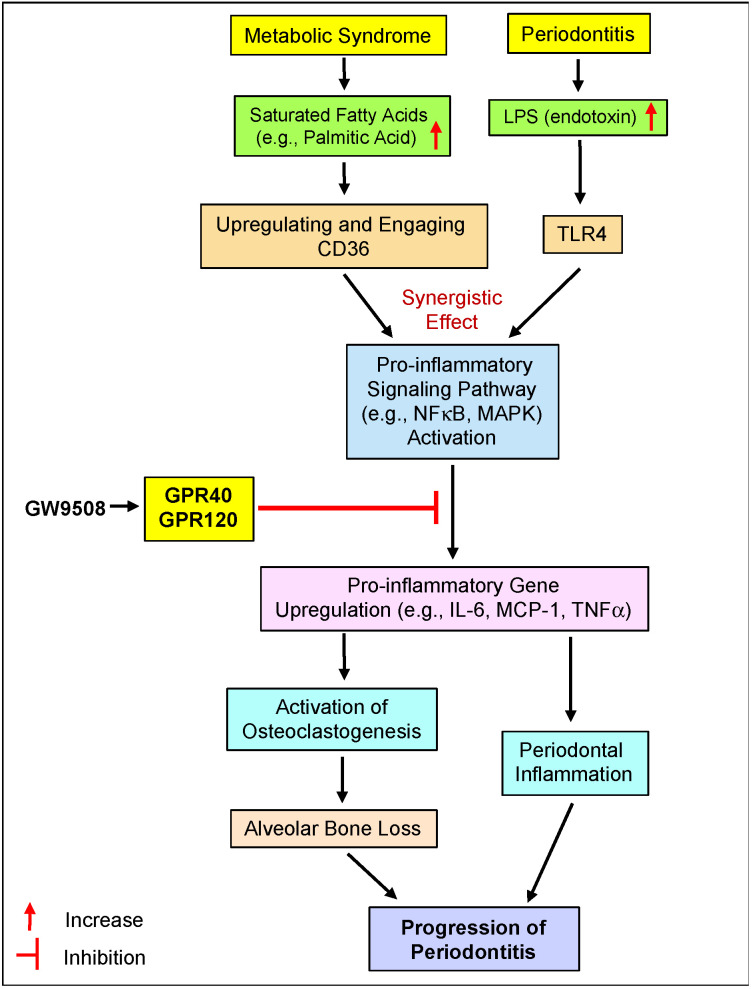
The potential mechanisms involved in the alleviation of alveolar bone loss and periodont inflammation by GPR40/GPR120 agonist GW9508 in mice with both MetS and periodontitis.

As illustrated in Figure 7, the current study showed, for the first time, that the synthetic dual GPR40/GPR120 agonist GW9508 alleviated alveolar bone loss and periodontal inflammation in mice with MetS-associated periodontitis. The investigations on the underlying mechanisms demonstrated that GW9508 inhibited osteoclastogenesis and inflammatory cytokine secretion stimulated by LPS and PA from macrophages. All these results strongly indicate that targeting GPR40/GPR120 with agonists is a potential anti-inflammatory approach in the treatment of MetS-associated periodontitis.

## 4. Materials and Methods

### 4.1. Animals

The Institutional Animal Care and Use Committee at the Medical University of South Carolina approved all experimental protocols. The study was performed in accordance with the Guidelines of ARRIVE. All mice were maintained on a 12-h light-dark cycle in a pathogen-free environment and had ad libitum access to water and food. MetS and periodontitis were induced by HFD feeding and periodontal LPS injection, respectively, and part of the mice were treated with GW9508 by intraperitoneal injection. Eight-week-old male C57BL/6 mice were randomly divided into 6 groups (n = 6): 1. Control; 2. Periodontitis; 3. Periodontitis with GW9508 treatment; 4. MetS; 5. MetS and periodontitis; 6. MetS and periodontitis with GW9508 treatment.

### 4.2. Induction of MetS

Mice were fed either LFD (D12450B) or HFD (D12492) (Research Diet Inc., New Brunswick, NJ, Canada) for 20 weeks. D12492 is a lard-based HFD containing 60% kcal fat, 20% kcal protein, and 20% kcal carbohydrate. For fat, it contains 254.5 g/kg fatty acid, 81.5 g/kg saturated fatty acid (SFA) and 51 g/kg PA. D12450B is a control LFD for D12492 containing 10% kcal fat, 20% kcal protein, and 70% kcal carbohydrate. For fat, it contains 43.7 g/kg fatty acid, 9.9 g/kg SFA and 1.1 g/kg PA.

### 4.3. Induction of Periodontitis and Treatment with GW9508

During the last 4 weeks of LFD or HFD feeding, some of the mice were injected with LPS isolated from *A.* actinomycetemcomitans (strain Y4, serotype B) [92] (0.2 mg/kg) through both left and right sides of the palatal gingiva between the maxillary 1st and 2nd molars, twice a week, for 4 weeks [93,94]. *A.* actinomycetemcomitans is one of the oral pathogens involved in chronic periodontitis [95]. For control, mice were periodontally injected with phosphate-buffered saline (PBS), the vehicle for LPS. Some of the mice were treated with GW9508 at 50 mg/kg/day by intraperitoneal injection (IP) during the last 4 weeks of LFD or HFD feeding when periodontal LPS injection was performed. The above dose of GW9508 was selected since it had been shown that treatment with GW9508 at the dose of 50 mg/kg/day through IP in C57BL/6 mice inhibited diabetes-induced neuropathy [96] and improved glucose homeostasis and insulin sensitivity [75]. The mice with IP of PBS were used as controls for GW9508 treatment.

### 4.4. Metabolic Measurements

Blood samples were obtained under the fasted condition, and glucose level was determined using a Precision QID glucometer (MediSense Inc., Bedford, MA, USA). Serum cholesterol was measured using the Cholestech LDX Lipid Monitoring System (Fisher Scientific, Pittsburgh, PA, USA). Serum triglycerides were measured using EnzyChrom™ Triglyceride Assay Kit (BioAssay Systems, Hayward, CA, USA). Serum FFAs were determined using the EnzyChrom™ free fatty acid kit (BioAssay systems (Hayward, CA, USA)). Serum fasting insulin was assayed using the Ultra Sensitive Insulin ELISA Kit (Crystal Chem, Inc., Downers Grove, IL, USA). Fasting whole-body insulin sensitivity was estimated with the HOMA-IR according to the formula [fasting plasma glucose (mg/dL) × fasting plasma insulin (mU/mL)]/405.

### 4.5. Micro-Computed Tomography (μCT), Analysis of Bone Volume Fraction (BVF), and Measurement of Cementoenamel Junction (CEJ)-Alveolar Bone Crest (ABC) Distance

Maxillae were dissected and fixed in 10% phosphate-buffered formalin for 24 h, washed with PBS, and stored in 70% ethanol. Maxillae were scanned at 55 kVp, 145 μA, 16 μm voxel resolution using Scanco Medical 40 mCT scanner (Scanco Medical, Brüttisellen, Switzerland) as described previously [97]. Three-dimensional images were generated and reconstructed for each specimen. These images were rotated with a standard orientation and threshold to discern mineralized and non-mineralized tissue. The region of interest (ROI) was indicated by the contour height of molars at the CEJ as the width and the molar cusp tips to root apices as the height. Depth was equal to the buccolingual size of the teeth plus 1.0 mm^3^. BVF was calculated as the percentage of bone within the ROI using AnalyzePro software (AnalyzeDirect, Inc., Overland Park, KS, USA), https://analyzedirect.com/analyzepro-support/ (accessed on 27 August 2024). A well-trained and experienced research specialist who was blinded to the treatment groups performed the mCT analysis. Data are reported in accordance with standardized nomenclature [98]. Additionally, three-line measurements of the distance from CEJ to ABC were taken for the first and second molars, as previously described [99].

### 4.6. Tartrate-Resistant Acid Phosphatase (TRAP) Staining and Quantification of Osteoclasts

Formalin-fixed maxillae were decalcified in a 10% EDTA solution for 4 weeks at 4 °C. The EDTA solution was changed three times per week. The maxillae were paraffin-embedded, and 7-μm sagittal sections were prepared. TRAP staining was performed in tissue sections using a leukocyte acid phosphatase kit (Sigma Aldrich, St. Louis, MO, USA). The tissue sections were counterstained with hematoxylin after TRAP staining. Pictures were taken with a Nikon Eclipse TS-100 inverted microscope. Active osteoclasts were defined as multinucleated TRAP-positive cells in contact with the bone surface. TRAP-positive osteoclasts under the first and the second molar on the surface of the alveolar bone were counted.

### 4.7. Histological Study of Periodontal Inflammation

For pathological evaluation, the above tissue sections were stained with hematoxylin and eosin. The tissue inflammation and bone resorption were evaluated according to the criteria for scoring tissue inflammation and bone resorption: 0 = within normal limits; 1 = focal some leukocyte infiltration, no significant bone resorption; 2 = moderate leukocyte infiltration with mild bone resorption; 3 = severe leukocyte infiltration with moderate bone resorption; and 4 = severe leukocyte infiltration with extensive bone resorption [100].

### 4.8. Cell Culture

RAW 264.7 macrophages were purchased from the American Type Culture Collection (Manassas, VA, USA) and grown in DMEM (American Type Culture Collection, Manassas, VA, USA) supplemented with 10% heat-inactivated fetal calf serum (HyClone, Logan, UT, USA). The cells were maintained in a 37 °C, 90% relative humidity, 5% CO_2_ environment.

### 4.9. PA Preparation

To prepare PA solution for cell treatments, PA (Sigma, St. Louis, MO, USA) was dissolved in 0.1 N NaOH and 70% ethanol at 70 °C to make PA with a stock concentration of 50 mM. The solution was kept at 55 °C for 10 min, mixed, and brought to room temperature.

### 4.10. Enzyme-Linked Immunosorbent Assay (ELISA)

Cytokines in the medium were quantified using a sandwich ELISA kit according to the protocol provided by the manufacturer (BioLegend, San Diego, CA, USA).

### 4.11. In Vitro Study of Osteoclast Formation

RAW264.7 macrophages were treated with 100 ng/mL of receptor activator of RANKL alone (control) or with RANKL plus 1 ng/mL of LPS, 100 mM of PA, or both LPS and PA for 3 days. The medium was then changed, and the macrophages were treated with RANKL alone or RANKL plus LPS, PA, or LPS plus PA in the absence or presence of 1 mM of GW9508 for 2 days. The RAW264.7 macrophages were treated with GW9508 during the last two days since it has been reported previously that significant changes in osteoclast differentiation induced by RANKL started on day 3 after RAW264.7 macrophages were exposed to RANKL [101]. After the treatment, cells were fixed with 4% paraformaldehyde in PBS for 10 min at room temperature. TRAP staining was carried out in accordance with the manufacturer’s instructions (Sigma Aldrich, St. Louis, MO, USA). Cells were observed under light microscopy, and those containing more than three nuclei were considered osteoclasts. The means of osteoclast number per well were counted and compared among different groups. The total area of multinuclear osteoclasts was measured with ImageJ software, version 2.0.0.-rc-43/1.52n (NIH, Bethesda, MD, USA) and presented as a % of the total area per field.

### 4.12. Statistical Analysis

GraphPad Prism 8 (v. 8.4.3) (GraphPad Software, Inc., La Jolla, CA, USA) was used for statistical analysis. The one-way analysis of variance (ANOVA) with the post hoc test was used to determine whether there were any statistically significant differences between the means of three or more independent groups. A Student’s *t*-test was performed for comparison between the two groups when data had a normal distribution. For data without normal distribution, nonparametric analysis using the Mann–Whitney test. For the in vitro study of osteoclast formation, the osteoclast numbers in three random fields per well viewed under the light microscope were counted, and the mean of the osteoclast numbers per well was calculated. The means of three wells were then compared between different groups. When an experiment was repeated, statistical analysis was also performed to compare their variances. The values were expressed as mean ± SD, and a value of *p* < 0.05 was considered significant.

## Figures and Tables

**Table 1:** The effect of GW9508 on bone volume fraction (BVF) in mice with periodontitis (PD) and/or metabolic syndrome (MetS).

	BVF (Mean SD)	% BVF Reduction Compared to Control Mice	% BVF Increase by GW9508 Treatment
Control	0.632 ± 0.014	-	
PD	0.583 ± 0.005	7.75%	
PD+GW9508	0.603 ± 0.013	4.59%	3.16%
MetS	0.530 ± 0.026	16.14%	
PD+MetS	0.470 ± 0.029	25.63%	
PD+MetS+GW9508	0.544 ± 0.012	13.92%	11.71%

## Data Availability

All data generated or analyzed during the current study are available from the corresponding author upon reasonable request.
